# Impact of *Paenarthrobacter ureafaciens* ZF1 on the soil enzyme activity and microbial community during the bioremediation of atrazine-contaminated soils

**DOI:** 10.1186/s12866-022-02556-4

**Published:** 2022-05-24

**Authors:** Zhifei Zhang, Qian Fu, Changyixin Xiao, Mingyue Ding, Dong Liang, Haitao Li, Rongmei Liu

**Affiliations:** 1 College of Life Sciences, Northeast Agricultural University, Harbin, 150030 China; 2 State Key Laboratory for Biology of Plant Diseases and Insect Pests, Institute of Plant Protection, Chinese Academy of Agricultural Sciences, Beijing, 10093 China

**Keywords:** Atrazine, Degrading strain ZF1, Bioremediation, Soil enzyme activity, Community structure

## Abstract

Bioremediation of atrazine-contaminated soil is considered a safe and effective approach in removing contaminates from the soil. However, the effects of adding foreign organisms to assist bioremediation on soil environmental quality and ecosystem are unclear. Here, the ecological remediation potential of strain *Paenarthrobacter ureafaciens* ZF1 on atrazine-contaminated soil was investigated through miniature experiments using variations in soil enzymes and bacterial communities as indicators. The results showed that strain ZF1 accelerated atrazine degradation, which could completely degrade atrazine at concentrations of 100 mg·L^− 1^ atrazine within 2 h in liquid medium and could remove up to 99.3% of atrazine (100 mg·kg^− 1^ in soil) within 6 days. During soil bioremediation, atrazine promoted the activities of urease and cellulase, and inhibited the activities of sucrase and catalase, while the strain ZF1 significantly promoted the activities of these four enzymes. High-throughput sequencing of 16S rRNA genes showed that ZF1 affected the relative abundance and bacterial community structure, and promoted bacterial diversity and evenness. Furthermore, redundancy analysis revealed a certain correlation among the strain ZF1, atrazine residue, soil enzyme activity, and soil bacterial community. The strain ZF1 in this work demonstrated remarkable potential for ecological restoration, and can be an effective and environmentally friendly alternative in remediating atrazine-contaminated soil.

**Supplementary Information:**

The online version contains supplementary material available at 10.1186/s12866-022-02556-4.

## Introduction

Atrazine (2-chloro-4-ethylamino-6-isopropylamino-1,3,5-triazine) is a triazine herbicide that exhibits high selectivity and endothermic properties. For the past 40 years, it has been widely used in controlling broad-leaved weeds and annual gramineous weeds growing in corn, wheat, sorghum, sugarcane, and other fields [1, 2]. However, due to its extended half-life, easy diffusion, and high-mobility, long-term use of atrazine may pollute the soil, surface water, and groundwater [3]. In addition, toxicological studies have revealed that low-level atrazine can interfere with the human endocrine system during long-term exposure, which poses carcinogenic or teratogenic risk [4]. Therefore, the problem associated with controlling atrazine pollution has received increasing attention in recent years. Microbial remediation of contaminated soil have become a popular choice because of their relatively low application cost and minimal environmental impact [5, 6]. At present, certain degrading bacteria have been isolated and applied in remediating atrazine – contaminated soil [7, 8], including strains of *Pseudomonas* sp. [9], *Rhodococcus* sp .[10], *Acinetobacter* sp. [11], *Arthrobacter* sp. [12], *Bacillus* sp. [13], *Agrobacterium* sp. [14] and *Glomus* sp. [15]. However, the biodegradation efficiencies of many bacteria were not efficient enough in most cases. It is necessary to isolate new bacterium which has much higher atrazine removal efficiency than the common bacteria. Additionally, the impact of these degrading strains on soil ecological environment during remediation is unclear. Therefore, it is necessary to scientifically and comprehensively monitor and evaluate the soil remediation process, soil environmental quality, and ecosystem variations.

Soil enzymes, which are closely associated with soil quality and nutrient circulation, are important components of the soil ecosystem [16]. Enzyme activities are considered to be sensitive to pollutants (such as heavy metals, pesticides, and crude oil, etc.), and their advantages include low cost and simple operation [17]. Chaudhary et al. [18] evaluated the impact of nanophos on soil enzyme activity, and found that soil enzyme activities were significantly improved in treated soil. In addition, Chaudhary et al. [19] also investigated the impact of nanozeolite and nanochitosan along with two *Bacillus* spp. on indicator enzymes to signify soil health underfield conditions on maize. The study revealed that the level of soil health indicator enzymes in the treatment group containing nanocompounds with *Bacillus* spp. up to two fold over control after 20, 40, and 60 days. Therefore, soil enzymes can also be used as indicators for assessing the quality of atrazine-contaminated soil [20]. In previous studies, the enzyme activities of sucrase, urease, cellulase, and catalase were selected to investigate the effects of long-term application of atrazine on soil health [21]. Among them, urease activity could measure the ability of soil microorganisms in catabolizing atrazine as a nitrogen source [22]. Furthermore, sucrase, cellulase, and catalase in soil are related to the carbon cycle and defense system [23, 24]. To date, there were many studies have focused on the effects of atrazine on soil enzyme activities [25–27]. However, the effects of degrading bacteria on soil enzyme activities during the remediation of atrazine have rarely been studied. Here in this work, soil enzymes as an indicator is taken into account to analyze the impact of exogenous degrading bacteria on soil health and soil ecological environment more comprehensively, which is helpful to deeply understand the relationship between soil enzymes and soil ecology.

As a key participant in various soil biochemical reactions, soil microorganisms usually participate in various soil life activities as one complete community. The change in community structure sensitively reflects the changes in soil quality, health status, and ecosystem [28–30]. Therefore, the change of microbial community structure can be used as an important ecological indicator to explore the impact of atrazine on soil quality and ecosystem [31]. Studies have shown that residual atrazine in soil considerably shifted the soil microbial community structure and function, and affected the growth of sensitive crops even at low concentrations [32, 33]. Hence, the remediation of atrazine-contaminated soil is particularly urgent and necessary. Hujiang et al. found that the application of atrazine reduced the bacterial community diversity, and the repair effect of degrading bacterium *Exiguobaterium* sp. BTAH1 restored the soil bacterial diversity [34]. *Arthrobacter* sp. strain HB-5 in the early stages of use, increased the quantities of indigenous bacteria, the microbial biomass carbon, and the Shannon, Simpson, and McIntosh diversity indices of soil microbes [35]. Strains *Chenggangzhangella methanolivorans* CHL1 and *Arthrobacter* sp. ART1 were applied to remediate chlorimuron-ethyl, atrazine, and a combination of contaminated soils; the results showed that inoculation treatments considerably relieved the effects of herbicides on soil microbial biomass, diversity, and community structure [36]. In conclusion, the ability of degrading bacteria in remediating atrazine-contaminated soil and its impact on soil ecological environment can be investigated by observing the changes in microbial community structure.

Herein, the bacterial strain *Paenarthrobacter ureafaciens* ZF1, which can completely degrade atrazine at concentrations of 100 mg·L^− 1^ atrazine within 2 h in liquid medium and remove up to 99.3% of atrazine (100 mg·kg^− 1^ in soil) within 6 days in soil, was isolated from severely polluted wheat farmland. The effect of ZF1 strain on soil microbial community and enzyme activities (sucrase, urease, cellulase, and catalase) during the bioremediation of atrazine-contaminated soils were investigated, as well as the correlation between strain ZF1, soil enzyme, atrazine residue, and bacterial community. This study provides a more efficient atrazine-degrading strain and enriches strain resources, which will fill the gap in the field of the impact of exogenous atrazine-degrading strains on soil enzyme activity and expand the understanding of the impact of degrading strains on bacterial community structure in the catabolism of pollutants, and also provide guidance for the in situ remediation of atrazine – contaminated soils.

## Methods

### Chemicals and media

Atrazine (purity: 99.8%) was purchased from Sinopharm Chemical Reagent Co., Ltd. (Shanghai, China). The soil enzyme kits were purchased from Suzhou Comin Biotechnology Co., Ltd. (Jiangsu, China). HPLC-grade methanol was used for HPLC analysis, and other chemicals used in this study were of analytical-grade. Mineral salt medium supplemented with atrazine (AMSM) was used to prepare cell enrichment culture [37].

### Sample collection and preparation

The soil used in the experiment was collected from the pesticide-free mountain forest near the farm in the suburbs of Harbin to ensure that it was not polluted by atrazine. The soil was air-dried, ground, and passed through a 2-mm sieve, and then divided into two portions. The first portion was artificially dosed with atrazine; atrazine (dissolved in methanol) was evenly sprayed such that its final concentration in soil was 100 mg·kg^− 1^. The soil mixture was dried in a fume hood to ensure complete volatilization of methanol. The second portion of the experimental soil was first sterilized (121 °C, 50 min), and then prepared similarly to that of the first portion.

### Experimental design and treatments

Strain ZF1 was inoculated in an AMSM enrichment medium, and cells grown until the OD_600_ value of 1.0, were harvested and washed thrice in sterile water. The enrichment culture was resuspended in sterile water to obtain a cell density of approximately 1 × 10^8^ colony forming units (CFU·mL^− 1^). One milliliter of treated bacteria was inoculated into the medium and Samples were taken every 0.25 h to test the degradation ability of strain ZF1 in liquid medium. The experimental treatments in soil considered were as follows: (A) sterilized soil (200 g) + sterile water (5 mL); (B) fresh soil (200 g) + sterile water (5 mL); (C) sterilized soil (200 g) + strain ZF1 (5 mL, 1 × 10^8^ CFU·mL^− 1^); and (D) fresh soil (200 g) + strain ZF1(5 mL, 1 × 10^8^ CFU·mL^− 1^). Each treatment was performed in triplicate, and all samples were cultured at room temperature (25 °C). During the test period, the soil-water content in each treatment was maintained at 20%. On day 2, 4, 6, 8, and 10, soil samples (10 g) were weighed from each group to detect the concentration of residual atrazine. The above treatment helped assess the remediation effect of strain ZF1 on atrazine-contaminated soil.

The experimental treatment methods similar to treatment groups (B) and (D) were used to explore the effect of strain ZF1 on soil enzyme activity and bacterial community. Samples were collected on days 2, 7, 14, and 28, and were divided into two portions. One subsample was used to analyze soil enzyme activities and the other was stored at − 80 °C for microbial community analysis. Moreover, treatment without atrazine or microbial inoculum was set as the blank control group (CK). Each treatment was performed in triplicate.

In microbial community analysis, the sample names are abbreviated as CK, AT1, AT2, AT3, AT4, ATJ1, ATJ2, ATJ3 and ATJ4. CK represents blank treatment, without atrazine and ZF1; AT represents only atrazine treated (100 mg·kg^− 1^); ATJ represents the atrazine treatment with strain ZF1. The numbers 1, 2, 3, and 4 represent 2, 7, 14, and 28 days of incubation, respectively. These samples were clustered into different groups according to their incubation time (2, 7, 14, and 28 days).

### Determination of atrazine in soil

Atrazine in soil was extracted with methanol. After extraction, aqueous samples were filtered through a 0.22 μm nylon filter before HPLC analysis. The concentration of atrazine in each sample was quantified using HPLC (Agilent, 1260 Infinity II) connected to a reverse-phase C18 column (4.6100 mm, 5 μm) and a variable – wavelength UV detector set to 215 nm. The flow rate was set to 1.0 mL·min^− 1^ (methanol /water = 80/20, v/v) and the column temperature was set to 30 °C.

According to the results of HPLC determination, the degradation rate of atrazine is calculated, and the percentage of atrazine degradation can be calculated as:

$$X=\frac{CCK- CX}{CCK}\times 100\%$$

where X is the degradation rate of atrazine; CX is the concentration of atrazine at different incubation times, mg·kg^− 1^; and CCK is the original concentration of atrazine, mg·kg^− 1^.

### Determination of soil enzyme activity

#### Determination of sucrase activity

Sucrase activity was determined by the 3,5-dinitrosalicylic acid colorimetric method [38]. Briefly, soil sample (0.1 g) was added to 15 μl toluene and the tubes were placed in water bath for 15 min at 37 °C. Phosphate buffer (250 μL) and sucrase (750 μL) was added. Tubes were placed in water bath (37 °C) for 24 h and centrifuged at 10000 g for 5 min at 4 °C. Supernatant (200 μL) was taken in another tube and DNS (500 μL) was added and placed in water bath at 95 °C for 5 min. Intensity of coloured product was measured by taking the readings at 510 nm after 10 times dilution with distilled water.

#### Determination of urease activity

The urease activity was analyzed by the indigo colorimetry method [16]. 0.25 g of soil sample and 125 μL toluene was taken in a tube, placed at room temperature for 15 minutes after mixing. Urea solution (625 μL) and citric acid buffer (1250 μL) was added. After incubation at 37 °C for 24 hours, entrifuged at 10000 g for 10 min at 25 °C and the supernatant was taken and diluted 10 times. 400 μL supernatant was taken in another tube and sodium phenol (80 μL) and sodium hypochlorite (60 μL) was added, fully mixed and placed at room temperature for 20 min. Finally, distilled water (460 μL) was added to dilute and activity was measured at a wavelength of 578 nm using a spectrophotometer.

#### Determination of cellulase activity

Soil cellulase activity was colorimetrically estimated by anthrone method [39]. 0.1 g soil sample and toluene (100 μL) was added in a test tube and the tube was shaken constantly for 15 min. Carboxymethylcellulase sodium solution (180 μL), acetic acid- anhydrous sodium acetate solution (740 μL) and distilled water (180 μL) were added in the tube. After shaking reaction at 37 °C for 3 h and placed in water bath at 90 °C for 15 min, entrifuged at 8000 g for 10 min at 25 °C and the supernatant was taken and get the saccharified liquid. Saccharified solution (350 μL) was taken in another tube and anthrone (650 μL) was added. Absorbance was taken at 620 nm after water bath at 90 °C for 10 min.

#### Determination of catalase activity

Soil catalase activity was determined by UV absorption method [38]. 0.1 g of soil and 1000 μL of H_2_O_2_ were added in a tube. Alum was added after shaking at 25 °C for 20 min. Supernatant (820 μL) was taken the after centrifuged at 8000 g for 5 min at 25 °C and sulfuric acid solution (96 μL) was added. Enzyme activity was calculated by taking the absorbance at 240 nm.

### Soil DNA extraction, PCR amplification and Illumina MiSeq sequencing

The total DNA extracted from soil samples was quantified by Nanodrop, and the quality of extracted DNA was detected using 1.2% agarose gel electrophoresis. The bacterial V3-V4 region of the 16S rRNA gene was amplified by Polymerase Chain Reaction (PCR) using the prokaryotic primers 338F (5′-ACTCCTACGGGAGGCA GCA-3′) and 806R (5′-GGACTACHVGGGTWTCTAAT-3′), and Pfu High-Fidelity DNA Polymerasesh [40]. The PCR products were purified using AxyPrep DNA Gel Recovery Kit according to the manufacturer’s instructions. Thereafter, a Microplate Reader (FLx800, BioTek), Quant-iT PicoGreen dsDNA Assay Kit, and TruSeq Nano DNA LT Library Prep Kit were used for fluorescent quantitative analysis and library constructions, and double-ended sequencing for deoxyribonucleic acid (DNA) of microbial composition was conducted using Illumina MiSeq.

The raw sequencing data were preliminarily screened, and the primers were removed, quality filtered, denoised, spliced, and chimaeras were removed according to DATA 2 [41]. DATA 2 no longer clusters with similarity, but only performs deduplication or its equivalent to clustering with 100% similarity. Each of the high-quality denoised sequences was termed as Amplicon Sequence Variant (ASV) or Operational Classifier Units (OTUs). The Greengenes database was used to compare the 16S rRNA bacterial genes and calculate the abundance of each taxon [42]. The information of sequencing parameters for different samples was available in Additional file: Table S6.

### Statistical analysis

The species composition at the phylum and genus levels, and the alpha diversity were analyzed using QIIME2 and R software. Chao1, Shannon, and Pielou indices (all Alpha Diversity Index) were used to evaluate the species richness, diversity, and evenness, respectively; the principal coordinate analysis (PCoA) was used to describe the differences between samples; Canoco 5 was used for redundancy analysis (RDA).

One-way analysis of variance (ANOVA) was used to analyze the significant differences between treatments, and *P* < 0.05 indicated that the differences were statistically significant. Origin 9.0 (Origin Lab, USA) was used for statistical computing and plotting.

## Results

### Degradation of atrazine

**Fig. 1:**
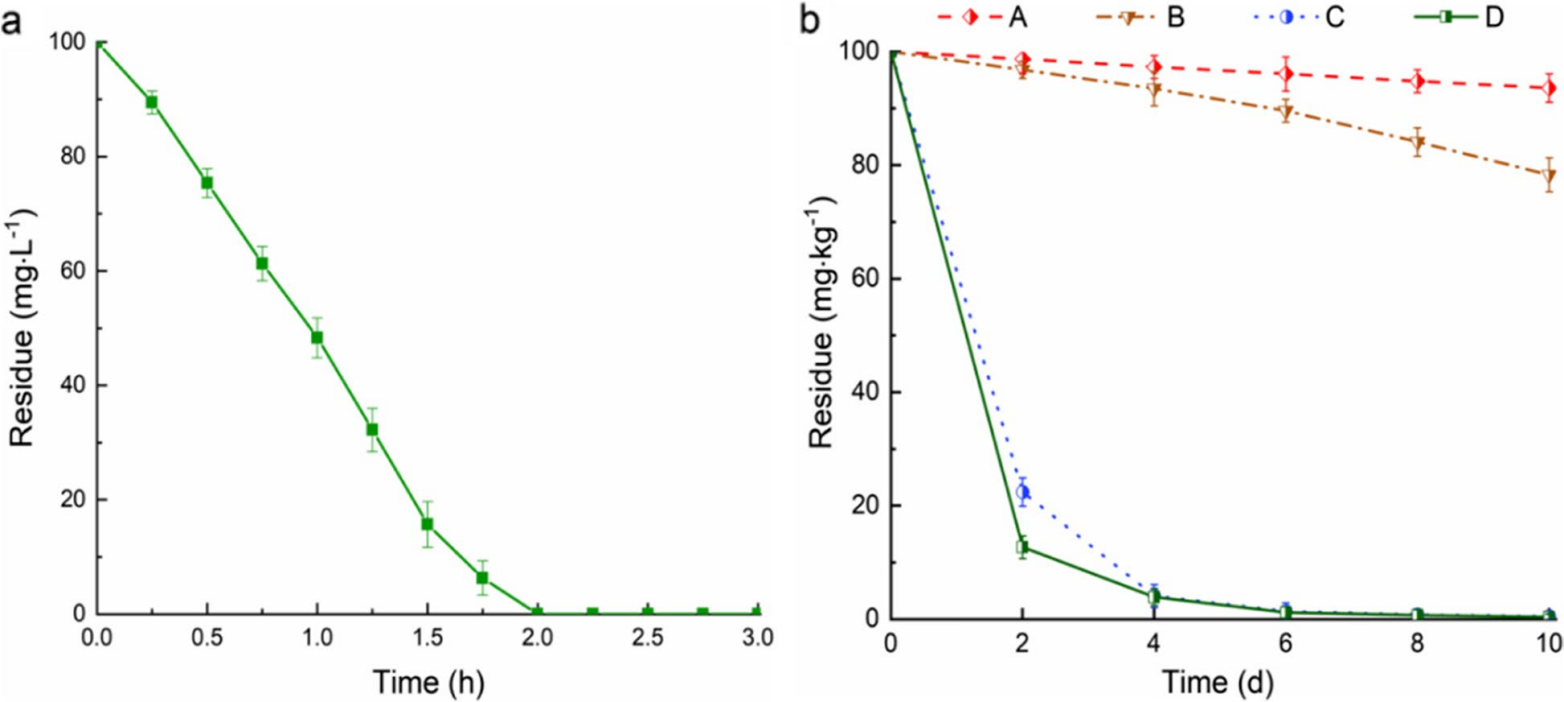
**a** Residues of atrazine in liquid medium; **b** Residues of atrazine in soil with treatments A-D during the incubation period. Treatment A: sterilized soil (200 g) + sterile water (5 mL); Treatment B: fresh soil (200 g) + sterile water (5 mL); Treatment C: sterilized soil (200 g) + strain ZF1 (5 mL, 1 × 10^8^ CFU·mL^− 1^); Treatment D: fresh soil (200 g) + strain ZF1 (5 mL, 1 × 10^8^ CFU·mL^− 1^)

Previously, the degradation of atrazine by strain ZF1 in an atrazine-containing mineral salts medium (concentration: 100 mg·L^− 1^) was tested and the results are given in Fig. 1a. Strain ZF1 completely degraded the atrazine at a concentration of 100 mg·L^− 1^ within 2 h, showing an excellent degradation efficiency compared to the degradation bacteria reported so far. Subsequently, we explored the ability of the strain ZF1 to degrade atrazine in soil. The residual dynamics of atrazine analyzed in four soil sample types (A, B, C, and D) are shown in Fig. 1b. The strain ZF1 significantly increased the degradation rate of atrazine in soil. After adding ZF1 to the soil, the degradation rates of atrazine (100 mg·kg^− 1^) reached 99.2 and 99.3% in 6 days in treatments (C) and (D), respectively. However, in soil without ZF1, the degradation rates reached only 3.9 and 10.4% in 6 days in treatments (A) and (B), respectively. Hence, the strain ZF1 remarkably affected the biodegradation of atrazine-contaminated soil. In addition, as shown in Fig. 1b, the degradation rate of atrazine in fresh soil was higher than that of sterilized soil, because certain microorganisms in fresh soil either directly participate in atrazine degradation or play auxiliary roles [43]. The amount of atrazine in the sterilized soil after 10 days was reduced by 6.4%, which may be attributed to hydrolysis or photolysis [44].

### Impact on soil enzyme activities

**Fig. 2:**
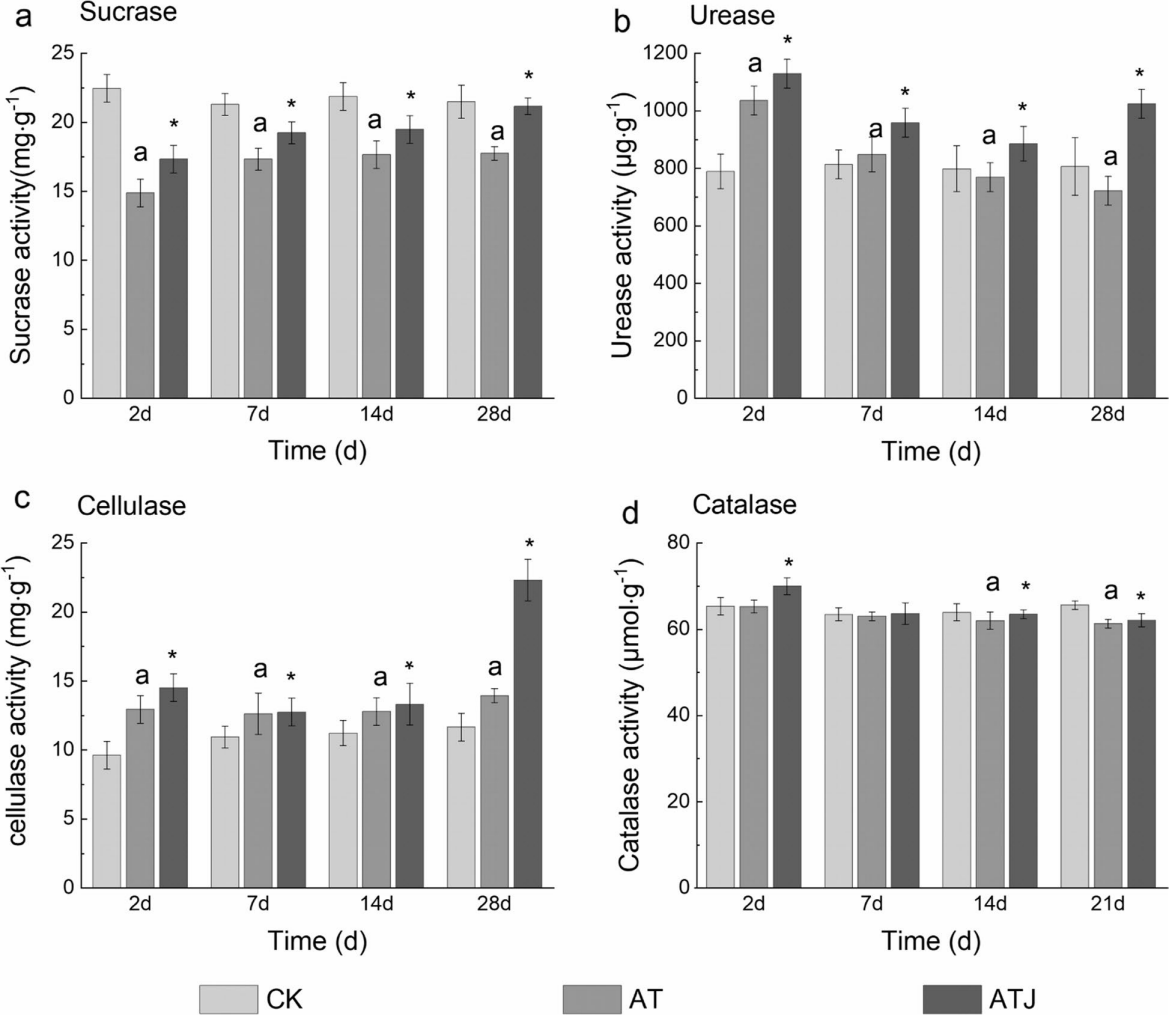
Effect of strain ZF1 on the activity of sucrase (**a**), urease (**b**), cellulase (**c**), and catalase (**d**) in soil treated with atrazine. The abscissa represents three soil types at different times: CK-soil without atrazine; AT-soil with atrazine (100 mg·kg^− 1^); ATJ-soil with strain ZF1 and atrazine (100 mg·kg^− 1^). For treatment without ZF1, significant differences between the atrazine treatment and CK group are marked with an “a” at *p* < 0.05. For treatment with atrazine, significant differences between treatment without ZF1 and ZF1-treated are marked with “*” at *p* < 0.05

As shown in Fig. 2a, atrazine (especially at high concentration) inhibited the sucrase activity in the soil. The application of strain ZF1 restored the inhibiting effect to the level in the control treatment and reduced the adverse effects of atrazine on the activities. In the CK group without atrazine, the activity of sucrase enzyme was 21.51 mg·g^− 1^. The day after the application of atrazine, the sucrase activities of AT (without ZF1treatment) group and ATJ (with ZF1 treatment) group were 14.9 mg·g^− 1^ and 17.36 mg·g^− 1^, respectively, which significantly decreased by 30.69 and 19.29%. With the degradation of atrazine in soil, the sucrase activities in AT and ATJ treatment groups gradually increased. On day 28, the sucrase activity in the ZF1-treated group reached 21.18 mg·g^− 1^, practically returning to the normal level, while the activity was only 17.77 mg·g^− 1^ in without ZF1 treated. In particular, the sucrase activity of ATJ group was always higher than AT group, indicating that strain ZF1 could promote the sucrase activity.

Figure 2b reveals that high concentration of atrazine (100 mg·kg^− 1^ of soil) could stimulate urease, while inoculation of strain ZF1 further improved soil urease activity. Briefly, for CK soil, the activity of urease was 789.83 μg·g^− 1^. On day 2 of atrazine application, urease activities were 1036.88 μg·g^− 1^ and 1129.82 μg·g^− 1^ under the conditions of without-ZF1 and ZF1-treated, respectively, which increased by 31.28 and 43.05% compared to that of CK. After a period of time, urease activity in AT and ATJ treatment groups decreased gradually with the decrease in atrazine concentration. At 28 days of incubation, the enzyme activity of AT group was lower than that of CK group, while that of ATJ was significantly higher than that in CK group.

Similar to soil urease, atrazine promoted the activity of cellulase, and the inoculated strain ZF1 further improved the enzyme activity (Fig. 2c). The activity in CK soil was 9.65 mg·g^− 1^. After incubating for 2 days, the cellulase activities of AT and ATJ group were 14.53 mg·g^− 1^ and 12.02 mg·g^− 1^, which significantly increased by 50.57 and 24.55%, respectively, compared to that of CK. Furthermore, on day 28, the cellulase activities of AT and ATJ treatment group significantly increased by 16.52 and 91.18%, respectively. During the entire incubation process, the cellulase activities of AT and ATJ treatment groups first notably increased, then decreased, and then increased again, but they were always higher than that of CK group, which indicates that atrazine could promote cellulase activity (especially the high levels).

As shown in Fig. 2d, atrazine inhibited catalase activities. The application of strain ZF1 improved the catalase activities and decreased the inhibiting effect of atrazine on the catalase activities in the soil. The catalase activity was 65.36 μmol·g^− 1^ without atrazine treated. After 2 days of atrazine treated, the catalase activity in AT treatment group remained unchanged compared to that of CK, while the enzyme activity in ZF1-treated group increased to 70.04 μmol·g^− 1^. However, with time, the enzyme activities of AT and ATJ treatment groups decreased slightly compared with that of CK group, but the enzyme activities of ATJ group were always higher than that of AT groups.

### Composition of bacteria communities and alpha diversity

**Fig. 3:**
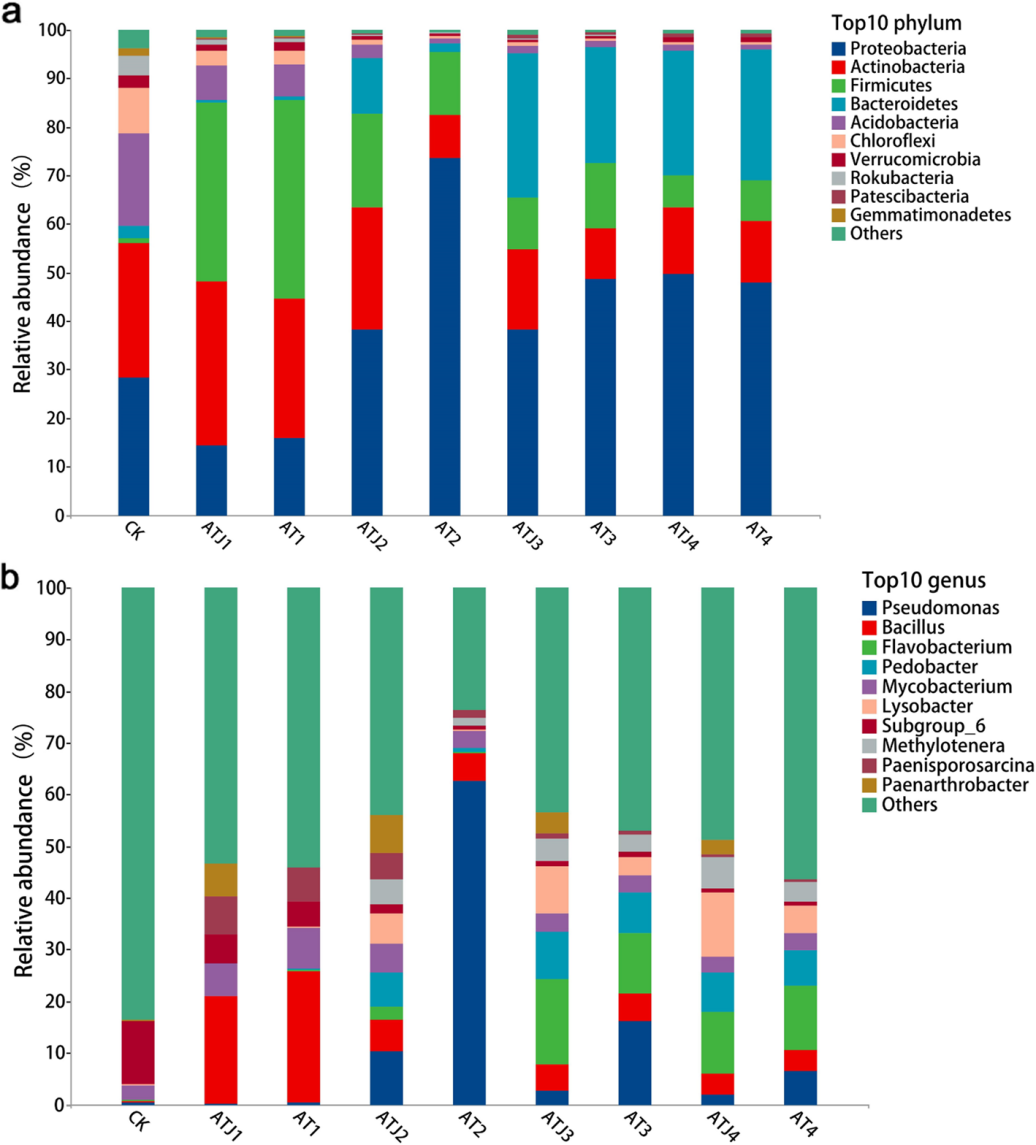
Relative abundance of soil bacterial community at (**a**) phylum level and (**b**) genus level. The top 10 predominant bacteria with relative abundance are shown at phylum and genus level, and the rest are merged into the others

Species taxonomy divides bacteria into seven classification levels, which are represented as domain, phylum, class, order, family, genus, and species [37]. The details about the relative abundance of different phylum, class, order, family, and genus level were provided in Additional file: Table S1-S5. Figure 3a and b represent the relative abundance of bacteria at phylum level and genus level, respectively. In the phylum classification level, the abundance of *Actinobacteria*, *Acidobacteria*, *Chloroflex*, and *Rokubacteria* gradually decreased after atrazine application. In addition, the abundance of *Firmicutes* increased first and then decreased, while the abundance of *Proteobacteria* and *Bacteroidetes* showed the opposite trend, which indicated that different levels of atrazine either promoted or inhibited the relative abundance of different phylum. In the later stages of incubation, the relative abundance of each phylum gradually stabilized and were dominated by *Proteobacteria* (47.95 -49.64%), *Bacteroidetes* (25.65-26.73%), *Actinobacteria* (12.67%-13.75), and *Firmicutes* (6.52-8.46%). In particular, in the ZF1-treated group, the abundance of *Actinobacteria* and *Bacteroidetes* increased compared to in no-ZF1 treated group, indicating that strain ZF1 affected the relative abundance of few bacteria to a certain extent.

For genus level classification, after treating with atrazine for some duration, the bacterial community structure mainly consisted of *Pseudomonas*, *Bacillus*, *Flavobacterium*, *Pedobacter*, *Mycobacterium*, *Lysobacter,* and *Methylenetra*. The relative abundance of species considerably varied during incubation. Among them, the abundance of *Flavobacterium* and *Pedobacter* largely increased in atrazine-treated soil, while the abundance of *subgroup*_6 largely decreased. Furthermore, the abundance of *Pseudomonas, Bacillus, Mycobacterium,* and *Paenisporosarcina* first increased and then decreased. The abundance of inoculated *Paenarthrobacter* gradually decreased with the increase of incubation time, because the available nutrient (atrazine) for *Paenarthrobacter ureafaciens* ZF1 decreased with incubation time. Thus, starvation might result in the decrease in the number of strain ZF1. Importantly, the addition of strain ZF1 significantly inhibited the abundance of *Pseudomonas* and promoted the abundance of *Lysobacter* and *Methylenetra*.

**Fig. 4:**
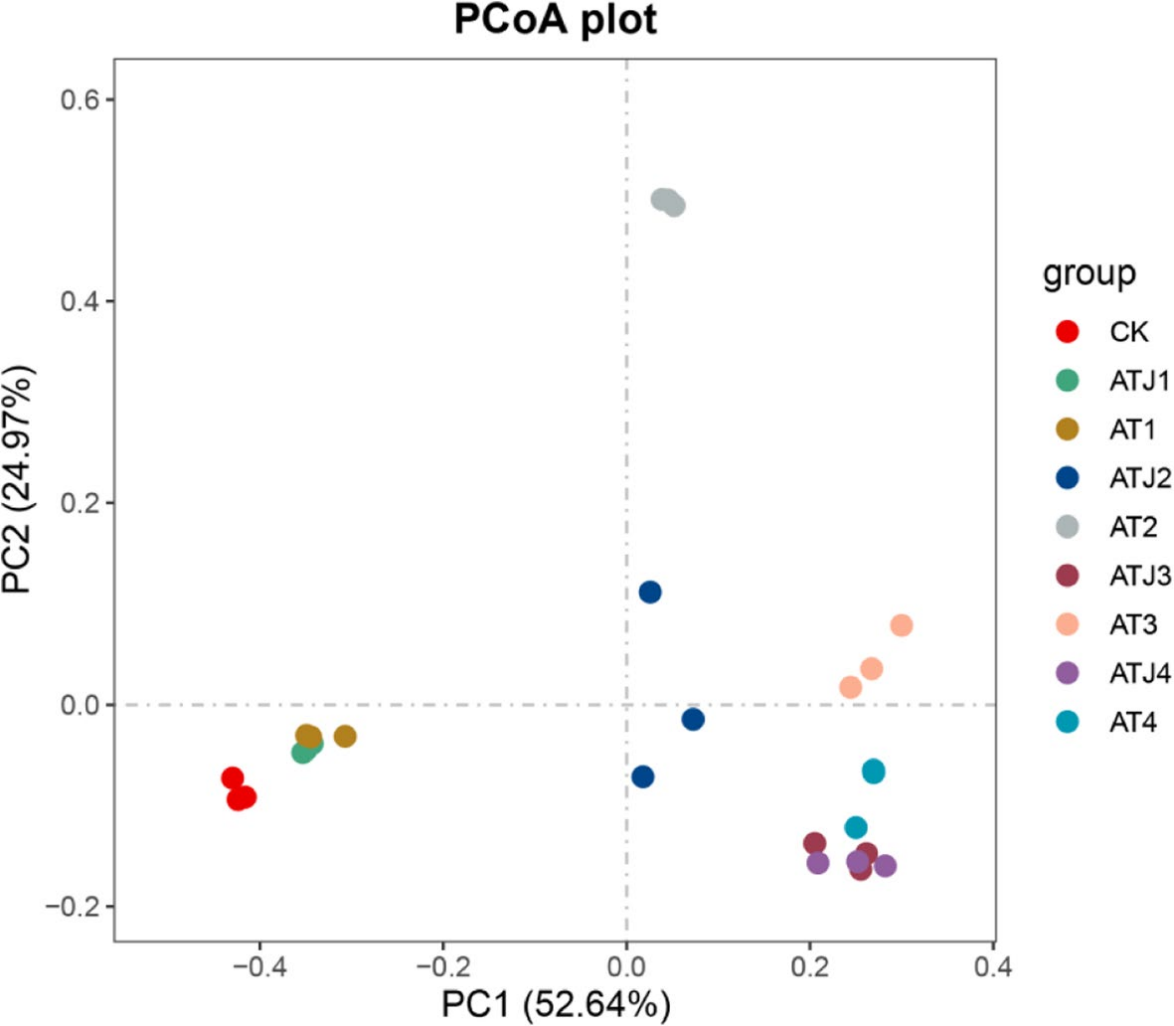
Principal coordinates analysis (PCoA) for the overall bacterial community composition of soil samples. The various colors indicate different samples; *n* = 3

Principal coordinates analysis (PCoA) for all treatment groups was performed according to the composition of OTUs in different treatment soils. As shown in Fig. 4, in the final experiment, the blank group (CK) significantly differed from the treatment group with atrazine (AT), indicating that atrazine altered the bacterial community structure of the soil. On day 2, AT1 and ATJ1 groups began to separate from CK group. On day 7, ATJ2 group (inoculated with ZF1) significantly differed from AT2 group (without ZF1), indicating that the community variation was the largest between the two treatments, and ATJ2 group was closer to other treatment groups in the later stages of incubation than that of AT2 group. The increase in incubation time shortened the distance between the two treatment groups. This shows that inoculating the strain ZF1 affected the bacterial community structure in soil to a certain extent, and helped achieve an immediate balance in the community structure.

Chao1, Pielou, and Shannon indices represent the relative richness, evenness and diversity of bacteria, respectively, in the soil habitat. As shown in Table 1, the presence of atrazine (especially high levels) in soil decreased the Chao1 index, which increased in the treatment group inoculated with strain ZF1. After 7 days of incubation, the Chao1 index of AT treatment group reached a minimum value of 2072, and the Chao1 index of the ATJ treatment group was 3422. With the increase in incubation time, the concentration of atrazine in soil gradually decreased, and the Chao1 index of AT groups increased gradually, while the increase in Chao1 index of the ZF1-treated ATJ groups were relatively slow. In particular, the total number of OTUs of bacteria in soil was consistent with the changing trend of the Chao1 index. Generally, atrazine could significantly inhibit the total number of bacterial OTUs and Chao1 index in soil, while the inoculation of strain ZF1 reduced the impact of atrazine on soil microorganisms and promoted the total number of OTUs and Chao1 index in its early stage of use. However, with the degradation of the substrate (atrazine), the amount of strain ZF1 decreased, which affected the recovery rate of bacterial number and the richness index of the soil bacterial community.

**Table 1:** The total number of OTUs of bacteria and Alpha diversity index

Sample	OTUs	Chao1	Pielou	Shannon
CK	3982	4131	0.920853	10.97
AT1	2942	3290	0.819581	9.45
ATJ1	3087	3480	0.804430	9.32
AT2	1693	2072	0.614152	6.59
ATJ2	2731	3422	0.770248	8.79
AT3	2284	2780	0.745753	8.32
ATJ3	2394	2926	0.771133	8.65
AT4	2918	3521	0.765136	8.79
ATJ4	2528	3040	0.782045	8.85

Similarly, Pielou index and Shannon index decreased first and increased after the application of atrazine. As shown in Table 1, on day 7, Pielou index and Shannon index reached the lowest at 0.614152 and 6.59, respectively, while the Pielou index and Shannon index of soil samples added with strain ZF1 increased to 0.770248 and 8.79, with an increase of 25.41 and 33.28%, respectively. Thus, in all atrazine spiked soil, strain ZF1 promoted Pielou index and Shannon index, indicating that adding ZF1 was beneficial in increasing the bacterial diversity and evenness.

### Correlation analysis between strain ZF1, soil enzyme, atrazine, and bacterial community

**Fig. 5:**
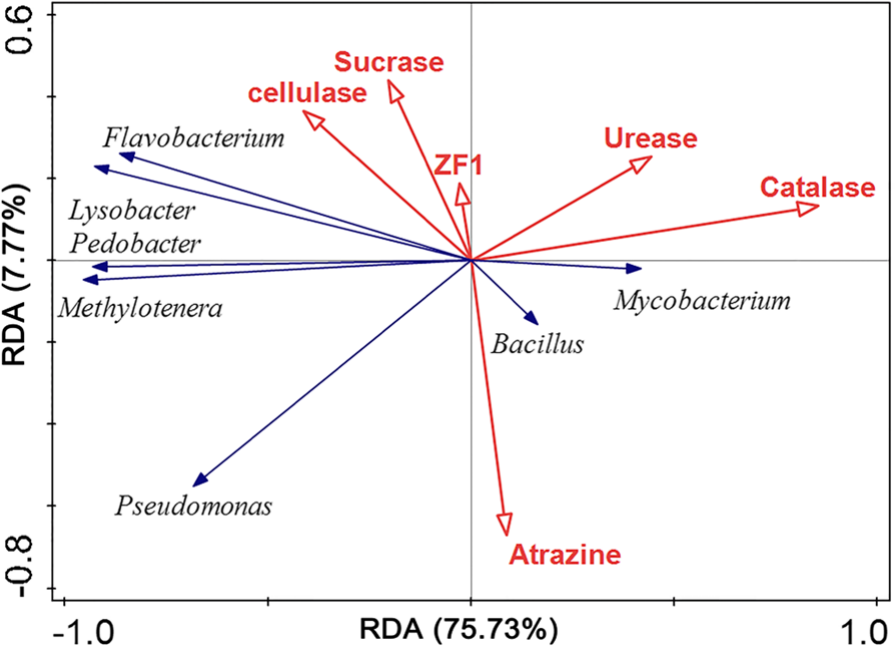
Redundancy analysis of the relationships among the abundance of strain ZF1, enzyme activities, atrazine residue, and bacterial genera

Redundancy analysis (RDA) was used to explore the relationship between the abundance of strain ZF1, soil enzyme activities, atrazine residue, and bacterial genera (soil dominant bacteria). Figure 5 shows that axis 1 and axis 2 explained 75.73 and 7.77% of the variability, respectively, accounting for 83.5% of the total variables. Among them, strain ZF1 positively correlated with *Flavobacterium* and *Lysobacter*, but negatively correlated with *Mycobacterium*, *Bacillus,* and *Pseudomonas*. In addition, strain ZF1 was positively correlated with four enzyme activities, and negatively correlated with atrazine residue. Cellulase and sucrase were positively correlated with *Flavobacterium*, *Lysobacter*; *Pedobacter* and *Methylenetra*, and negatively correlated with *Mycobacterium* and *Bacillus*. Urease and catalase were positively correlated with *Mycobacterium* and *Bacillus*, and negatively correlated with the other five bacterial genera. Atrazine was positively correlated with *Bacillus, Mycobacterium*, and *Pseudomonas*, and negatively correlated with the other four genera and four enzymes.

## Discussion

### Residual dynamics of atrazine in soil

Soil and water pollution by atrazine is a persistent problem worldwide, and poses a considerable threat to the health of soil ecosystem, and adversely influences the soil biological processes by altering soil enzymatic activities, soil microbial population and community diversity [45–47]. Therefore, it is crucial to find a practical solution for the remediation of atrazine -contaminated environments. Atrazine in soil can be degraded by both biotic and abiotic processes. Among them, microbial-assisted treatment method is ideal for treating atrazine residues, as it is more cost-effective and environmentally friendly than other methods [48]. This study simulated the outdoor soil environment and reported the remediation potential of strain ZF1 for atrazine-contaminated soil, which could degrade 99.3% atrazine (100 mg·kg^− 1^) in soil in 6 days. In previous reports, after 7 days of LY-1 incubation, atrazine (100 mg·kg^− 1^) in soil was removed by 79.9% [49]; *Paenarthrobacter* sp. AT − 5 could remove 95.9% of 50 mg·kg^− 1^ atrazine was from the soils with 7 days [50]; after 6 days of treatment using strain YQJ-6, the degradation rates of atrazine (50 mg·kg^− 1^) in fresh soil and sterilized soil were 99.8 and 95.2%, respectively [51]. Strain ZF1 could degrade higher concentration of atrazine in soil in practically the same time, exhibiting it had greater atrazine-degrading ability than most atrazine-degrading microorganisms. Therefore, the strain ZF1 can be a good candidate for the remediation of atrazine-contaminated soil.

### Effects of atrazine and ZF1 on soil enzyme activities

In soil ecosystem, soil enzymes play an important role in maintaining soil structure, circulating nutrients, and detoxifying pollutants, and help catalyze most biochemical reactions in soil [52]. Soil enzyme activity can reflect the health status of soil and is used to evaluate the impact of herbicides on soil ecological environment [53]. In this study, four soil enzymes presented different responses to atrazine. Among them, atrazine inhibited the activities of sucrase and catalase, but activated soil urease and cellulase. Most soil enzymes are extracellular metabolites secreted by soil [54, 55]. The application of atrazine altered the structure and abundance of soil microbial community, thus affecting the activity of soil enzymes [21]. In addition, the degradation products of atrazine can provide substrate for urease, consecutively affecting urease activity [56]. The addition of strain ZF1 uniformly promoted the activities of sucrase, urease, cellulase, and catalase in soil. Redundancy analysis also showed that ZF1 was positively correlated with these four enzymes, indicating that strain ZF1 could improve the biochemical properties and quality of the soil. Nevertheless, specific mechanism followed by strain ZF1 in promoting soil enzyme activity needs to be further studied.

### Effects of atrazine and ZF1 on soil microbial community

Microorganisms are extremely sensitive to changes in their surroundings and appear to appropriately predict soil health and monitor soil ecological environment [57]. Therefore, changes in soil microbial community structure was selected to investigate the impact of strain ZF1 on soil ecological environment. According to the composition spectrum analysis of microbial community diversity, the composition of bacterial community changed significantly after the application of atrazine, which was consistent with the previously reported results [13]. At the phyla level, the inoculation of strain ZF1 increased the abundance of *Actinobacteria* and *Bacteroidetes* in the community, indicating that they were positively correlated with strain ZF1; Similarly, at the genus level, strain ZF1 promoted the abundance of some beneficial beneficial bacteria such as *Lysobacter* in polluted soil, so ZF1 was positively correlated with *Lysobacter*. These bacterial phyla or genera may promote the growth of ZF1 to improve the degradation rate of atrazine [33]. In addition, the results of PCoA analysis suggested that the application of atrazine affected the community structure, and the inoculation of strain ZF1 accelerated the community succession and restored the balance of the community structure faster. This may be because the bacteria in the microbial inoculum degraded the pollutants and reduced the influence of pollutants on the soil microorganisms, consequently restoring the community structure of microorganisms in the soil [58]. Microbial community diversity analysis indicated that strain ZF1 increased Pielou index and Shannon index, thereby promoting the diversity and evenness of the bacterial community in atrazine-contaminated soil, and displayed good ecological benefits. Therefore, the inoculation of strain ZF1 has very important ecological significance for the remediation of atrazine-contaminated soil.

In addition, the redundancy analysis results showed the relative abundance of *Flavobacterium* and *Lysobacter* were positively correlated with strain ZF1, which many enhance atrazine degradation via promoting the growth of ZF1. Furthermore, *Bacillus*, *Mycobacterium*, and *Pseudomonas* showed a positive correlation with atrazine residue, as they suppressed atrazine degradation directly or indirectly. These results suggested that atrazine-degrading efficiency was affected by not only the inoculated bacteria but also some other bacterial genera in the community. The promotion and suppression of atrazine degradation by the bacteria in the community, showing that shifts in both the composition and abundance of these bacteria genera can drive the change in the efficiency of catabolic function [33]. Some studies have also shown that the presence of some bacteria might alter the proportion of some organic components (such as the C/N ratio) in the environment, thereby affecting the degradation of atrazine [59]. Besides, *Flavobacterium* and *Lysobacter* were positively correlated with cellulase and sucrase, *Mycobacterium* and *Bacillus* were positively correlated with urease and catalase, as they may secrete these enzymes themselves or promote the secretion of these enzymes by other bacteria. On the contrary, *Pseudomonas* and other bacteria genera were negatively correlated with these four enzymes, indicating that these bacteria had inhibitory effects on these enzymes. In conclusion, there were certain correlations among strain ZF1, soil enzymes, and bacterial communities, indicating that ZF1 affects the soil enzyme activities and bacterial communities. A certain correlation between soil microorganisms and soil enzymes was also observed, which indicated that soil microorganisms affected soil enzyme activities. In contrast, soil enzymes also affected the species and bacterial abundance. In this paper, degradation bacteria, atrazine residues, soil enzymes, and community structure are linked together through redundancy analysis to clarify the relationship between these four factors, making the study more complete and systematic.

## Conclusion

The study simulated a microbial remediation experiment in the laboratory to explore the ability of strain ZF1 in atrazine-contaminated soil. The results showed that strain ZF1 could remove 99.3% atrazine (100 mg·kg^− 1^) from soil in 6 days, and exhibited good biodegradability. During soil remediation, sucrase, urease, cellulase, and catalase responded differently to atrazine, and the inoculation of strain ZF1 promoted the activities of these four enzymes. In addition, strain ZF1 affected the bacterial abundance and community structure, and accelerated the succession of community to a certain extent. Pielou index and Shannon index revealed that strain ZF1 promoted bacterial diversity and uniformity. Redundancy analysis revealed a certain correlation among degrading bacterium ZF1, atrazine residue, soil enzyme activity, and soil bacterial community; they affected each other and were interdependent. In general, strain ZF1 was conducive in promoting the soil ecological function during atrazine pollution, reduced the adverse impact of atrazine on soil health, and exhibited remarkable ecological restoration potential. This study provides a novel highly efficient candidate for atrazine bioremediation and enriches strain resources, and also provides useful information for the design of bioremediation strategies for contaminated soil.

## Supplementary Information

**Additional file 1: Table S1.** Relative abundance of the top 10 predominant bacteria in soil bacterial community at phyla level. **Table S2.** Relative abundance of the top 10 predominant bacteria in soil bacterial community at class level. **Table S3.** Relative abundance of the top 10 predominant bacteria in soil bacterial community at order level. **Table S4.** Relative abundance of the top 10 predominant bacteria in soil bacterial community at family level. **Table S5.** Relative abundance of the top 10 predominant bacteria in soil bacterial community at genus level. **Table S6.** Information of sequencing parameters for different samples.

## Data Availability

The datasets generated and/or analysed during the current study are available in the GenBank (http://www.ncbi.nlm.nih.gov/genbank) under Accession number OL415511. High throughput analysis data generated from this study have been deposited in NCBI SRA (https://www.ncbi.nlm.nih.gov/sra) under the accession number PRJNA835293.

## References

[1] Ma L, Chen S, Yuan J, Yang P, Liu Y, Stewart K (2017). Rapid biodegradation of atrazine by *Ensifer* sp. strain and its degradation genes. Int Biodeterior Biodegradation.

[2] Bhardwaj P, Singh KR, Jadeja NB, Phale PS, Kapley A (2020). Atrazine bioremediation and its influence on soil microbial diversity by metagenomics analysis. Indina J Microbiol.

[3] Delwiche KB, Lehmann J, Walter MT (2014). Atrazine leaching from biochar-amended soils. Chemosphere.

[4] Siripattanakul S, Wirojanagud W, McEvoy J, Limpiyakorn T, Khan E (2009). Atrazine degradation by stable mixed cultures enriched from agricultural soil and their characterization. J Appl Microbiol.

[5] Rehan M, Kluge M, Fraenzle S, Kellner H, Ullrich R, Hofrichter M (2014). Degradation of atrazine by *Frankia alni ACN14a*: gene regulation, dealkylation, and dechlorination. Appl Microbiol Biot.

[6] Nousiainen AO, Bjorklof K, Sagarkar S, Nielsen JL, Kapley A, Jorgensen KS (2015). Bioremediation strategies for removal of residual atrazine in the boreal groundwater zone. Appl Microbiol Biot.

[7] Hu J, Dai X, Li S (2005). Bioemediation of atraine in unsterilized soil by two atrazine degradation strains. Acta Pedol Sin.

[8] Ofaim S, Zarecki R, Porob S, Gat D, Lahav T, Kashi Y (2020). Genome-scale reconstruction of *Paenarthrobacter aurescens* TC1 metabolic model towards the study of atrazine bioremediation. Sci Rep.

[9] Fernandes AFT, da Silva MBP, Martins VV, Martins VV, Miranda CES, Stehling EG (2014). Isolation and characterization of a *Pseudomonas aeruginosa* from a virgin Brazilian Amazon region with potential to degrade atrazine. Environ Sci Pollut Res.

[10] Fazlurrahman BM, Pandey J, Suri CR, Jain RK (2010). Isolation and characterization of an atrazine-degrading *Rhodococcus* sp. strain MB-P1 from contaminated soil. Lett Appl Microbiol.

[11] Wang ZG, Zhang Y, Guo HS, Huan Y (2014). Response of an atrazine-degrading bacterium strain *Acinetobacter* sp. DNS32 to inorganic nitrogen source. Microbiol China.

[12] Zhang Y, Jiang Z, Cao B, Hu M, Wang Z, Dong X (2011). Metabolic ability and gene characteristics of *Arthrobacter* sp. strain DNS10, the sole atrazine-degrading strain in a consortium isolated from black soil. Int Biodeterior Biodegradation.

[13] Kolekar PD, Patil SM, Suryavanshi MV, Suryawanshi SS, Khandare RV, Govindwar SP (2019). Microcosm study of atrazine bioremediation by indigenous microorganisms and cytotoxicity of biodegraded metabolites. J Hazard Mater.

[14] Struthers JK, Jayachandran K, Moorman TB (1998). Biodegradation of atrazine by *Agrobacterium radiobacter* J14a and use of this strain in bioremediation of contaminated soil. Appl Environ Microb.

[15] Song F, Ding M, Dong A, Fan X, Zhao X (2010). Effect of arbuscular mycorrhizal (AM) fungi on atrazine degradation in soil planted sorghum. J Soil Water Conserv.

[16] Chen J, Yang WP, Li J, Anwar S, Wang K, Yang ZP (2021). Effects of herbicides on the microbial community and urease activity in the rhizosphere soil of maize at maturity stage. J Sensors.

[17] Lee SH, Kim MS, Kim JG, Kim SO (2020). Use of soil enzymes as indicators for contaminated soil monitoring and sustainable management. Sustainability.

[18] Chaudhary P, Chaudhary A, Parveen H, Rani A, Kumar G, Kumar R (2021). Impact of nanophos in agriculture to improve functional bacterial community and crop productivity. BMC Plant Biol.

[19] Chaudhary P, Chaudhary A, Bhatt P, Kumar G, Khatoon H, Rani A (2022). Assessment of soil health indicators under the influence of nanocompounds and *Bacillus* spp. in field condition. Front Env Sci.

[20] Ling D, Huang Q, Ouyang Y (2010). Impacts of simulated acid rain on soil enzyme activities in a latosol. Ecotoxicol Environ Saf.

[21] Liu YF, Fan XX, Zhang T, He WY, Song FQ (2020). Effects of the long-term application of atrazine on soil enzyme activity and bacterial community structure in farmlands in China. Environ Pollut.

[22] Dewey KA, Gaw SK, Northcott GL, Lauren DR, Hackenburg S (2012). The effects of copper on microbial activity and the degradation of atrazine and indoxacarb in a New Zealand soil. Soil Biol Biochem.

[23] Kaya C, Akram NA, Surucu A, Ashraf M (2019). Alleviating effect of nitric oxide on oxidative stress and antioxidant defence system in pepper (*Capsicum annuum L.*) plants exposed to cadmium and lead toxicity applied separately or in combination. Sci Hortic.

[24] Yang F, Yang S, Xu J, Wang Y, Gao M, Zhang M (2021). Dynamic response of soil enzymes and microbial diversity to continuous application of atrazine in black soil of a cornfield without rotation in Northeast China. Diversity.

[25] Xin C, Zhu L, Wang J, Sun R, Zhao B (2004). Effect of atrazine on soil invertase under different soil fertilities. J Agro Environ Sci.

[26] Mahia J, Gonzalez-Prieto SJ, Martin A, Baath E, Diaz-Ravina M (2011). Biochemical properties and microbial community structure of five different soils after atrazine addition. Biol Fertil Soils.

[27] Zhang Y, Cao B, Jiang Z, Zhang X, Wang Z, Li R (2017). Effect of atrazine on soil enzyme activities and microbial community structure in black soil. J Northeast Agric Univ.

[28] Teng Y, Huang C, Luo Y, Long J, Yao H (2004). Microbial activitues and functional diversity of community in soils polluted with Pb-Zn-ac mine tailings. Acta Pedol Sin.

[29] Xu J, Hu N, Zhang Z, Tao B, Zhu L (2015). Effects of two herbicides on soil microbes and enzyme activities in a paddy field. Bull Soil Water Conserv.

[30] Yang L, Jiang M, Zhu W, Hand L, Qina L (2019). Soil bacterial communities with an indicative function response to nutrients in wetlands of northeastern China that have undergone natural restoration. Ecol Indic.

[31] An X, Wang Z, Teng X, Zhoub R, Wang X, Xua M (2022). Rhizosphere bacterial diversity and environmental function prediction of wild salt-tolerant plants in coastal silt soil. Ecol Indic.

[32] Bach EM, Baer SG, Meyer CK, Six J (2010). Soil texture affects soil microbial and structural recovery during grassland restoration. Soil Biolo Biochem.

[33] Fang H, Lian J, Wang H, Cai L, Yu Y (2015). Exploring bacterial community structure and function associated with atrazine biodegradation in repeatedly treated soils. J Hazard Mater.

[34] Hu J, Dai X, Li S (2005). Effects of atrazine and its degrader *Exiguobaterium* sp. BTAH1 on soil microbial community. Ying Yong Sheng Tai Xue Bao.

[35] Gao J, Song P, Wang G, Wang J, Zhu L, Wang J (2018). Responses of atrazine degradation and native bacterial community in soil to *Arthrobacter* sp. strain HB-5. Ecotox Environ Safe.

[36] Wang J, Li X, Li X, Wang H, Su Z, Wang X (2018). Dynamic changes in microbial communities during the bioremediation of herbicide (chlorimuron-ethyl and atrazine) contaminated soils by combined degrading bacteria. PLoS One.

[37] Li Y, Zhang J, Sha J, Shi C, Chi B, Gao J (2018). Isolation and identification of atrazine-degrading bacterial strain LY-2 and its bioremediation to contaminated soil. J Agri Biotechnol.

[38] Cai M, Hu C, Wang X, Zhao Y, Jia W, Sun X (2019). Selenium induces changes of rhizosphere bacterial characteristics and enzyme activities affecting chromium/selenium uptake by pak choi (*Brassica campestris L.* ssp. *Chinensis Makino*) in chromium contaminated soil. Environ Pollut.

[39] Yu P, Tang X, Zhang A, Fan G, Liu S (2019). Responses of soil specific enzyme activities to short-term land use conversions in a salt-affected region, northeastern China. Sci Total Environ.

[40] Xu L, Han Y, Yi M, Yi H, Guo E, Zhang A (2019). Shift of millet rhizosphere bacterial community during the maturation of parent soil revealed by 16S rDNA high-throughput sequencing. Appl Soil Ecol.

[41] Callahan BJ, McMurdie PJ, Rosen MJ, Han AW, Johnson AJA, Holmes SP (2016). DADA2: high-resolution sample inference from Illumina amplicon data. Nat Methods.

[42] DeSantis TZ, Hugenholtz P, Larsen N, Rojas M, Brodie EL, Keller K (2006). Greengenes, a chimera-checked 16S rRNA gene database and workbench compatible with ARB. Appl Environ Microbiol.

[43] Douglass JF, Radosevich M, Tuovinen OH (2017). Microbial attenuation of atrazine in agricultural soils: biometer assays, bacterial taxonomic diversity, and catabolic genes. Chemosphere.

[44] Parra S, Stanca SE, Guasaquillo I, Thampi KR (2004). Photocatalytic degradation of atrazine using suspended and supported TiO_2_. Appl Catal B Environ.

[45] Song R, Liu L, Wu C, Ma L (2009). Effect of atrazine on chernozem soil microbial activity in semiarid region of Northeast China. J Agro Environ Sci.

[46] Chen Q, Wang H, Yang B, He F (2014). The combined effects of atrazine and lead (Pb): relative microbial activities and herbicide dissipation. Ecotox Environ Safe.

[47] Liu X, Chen K, Chuang S, Xu X, Jiang J (2019). Shift in bacterial community structure drives different atrazine-degrading efficiencies. Front Microbiol.

[48] Satsuma K (2010). Mineralization of s-triazine herbicides by a newly isolated *Nocardioides* species strain DN36. Appl Microbiol Biot.

[49] Li Y, Liang D, Sha J, Zhang J, Gao J, Li H (2019). Isolating and identifying the atrazine-degrading strain *Arthrobacter* sp. LY-1 and applying it for the bioremediation of atrazine-contaminated soil. Pol J Environ Stud.

[50] Jia W, Li N, Yang T, Dai W, Jiang J, Chen K (2021). Bioaugmentation of atrazine-contaminated soil with Paenarthrobacter sp. strain AT-5 and its effect on the soil microbiome. Front Microbiol.

[51] Zhu J, Fu L, Meng Z, Jin C (2021). Characteristics of an atrazine degrading bacterium and the construction of a microbial agent for effective atrazine degradation. Water Environ J.

[52] Alkorta I, Aizpurua A, Riga P, Albizu I, Amezaga I, Garbisu C (2003). Soil enzyme activities as biological indicators of soil health. Rev Environ Health.

[53] Pan J, Yu L (2011). Effects of cd or/and Pb on soil enzyme activities and microbial community structure. Ecol Eng.

[54] Liu YM, Cao WQ, Chen XX, Yu BG, Lang M, Chen XP (2020). The responses of soil enzyme activities, microbial biomass and microbial community structure to nine years of varied zinc application rates. Sci Total Environ.

[55] Yang R, Wang J, Zhu L, Wang J, Yang L, Mao S (2021). Effects of interaction between enrofloxacin and copper on soil enzyme activity and evaluation of comprehensive toxicity. Chemosphere.

[56] Moreno JL, Aliaga A, Navarro S, Hernandez T, Garcia C (2007). Effects of atrazine on microbial activity in semiarid soil. Appl Soil Ecol.

[57] Epelde L, Becerril JM, Kowalchuk GA, Deng Y, Zhou J, Garbisu C (2010). Impact of metal pollution and Thlaspi caerulescens growth on soil microbial communities. Appl Environ Microbiol.

[58] Subashchandrabose SR, Ramakrishnan B, Megharaj M, Venkateswarlu K, Naidu R (2013). Mixotrophic cyanobacteria and microalgae as distinctive biological agents for organic pollutant degradation. Environ Int.

[59] Abdelhafid R, Houot S, Barriuso E (2000). Dependence of atrazine degradation on C and N availability in adapted and non-adapted soils. Soil Biol Biochem.

