# Supplementary material for: Impact of Paenarthrobacter ureafaciens ZF1 on the soil enzyme activity and microbial community during the bioremediation of atrazine-contaminated soils
Source: BMC Microbiol. 2022 May 24;22:146. doi: 10.1186/s12866-022-02556-4 (PMC9128208; doi:10.1186/s12866-022-02556-4)
Supplement: Supplementary file 1 — Additional file 1: Table S1. Relative abundance of the top 10 predominant bacteria in soil bacterial community at phyla level. Table S2. Relative abundance of the top 10 predominant bacteria in soil bacterial community at class level. Table S3. Relative abundance of the top 10 predominant bacteria in soil bacterial community at order level. Table S4. Relative abundance of the top 10 predominant bacteria in soil bacterial community at family level. Table S5. Relative abundance of the top 10 predominant bacteria in soil bacterial community at genus level. Table S6. Information of sequencing parameters for different samples. [file 12866_2022_2556_MOESM1_ESM.docx]

**Additional Information**

**Table S1.** Relative abundance of the top 10 predominant bacteria in soil bacterial community at phyla level

| Phyla | CK | ATJ1 | AT1 | ATJ2 | AT2 | ATJ3 | AT3 | ATJ4 | AT4 |
| --- | --- | --- | --- | --- | --- | --- | --- | --- | --- |
| *Proteobacteria* | 0.283612 | 0.143065 | 0.159124 | 0.381611 | 0.736644 | 0.382607 | 0.486918 | 0.496475 | 0.479599 |
| *Actinobacteria* | 0.277759 | 0.339332 | 0.287271 | 0.252870 | 0.088946 | 0.164809 | 0.102834 | 0.137503 | 0.126782 |
| *Firmicutes* | 0.008516 | 0.367857 | 0.408827 | 0.192596 | 0.128785 | 0.106706 | 0.134918 | 0.065277 | 0.084648 |
| *Bacteroidetes* | 0.024877 | 0.003827 | 0.007800 | 0.114965 | 0.016898 | 0.296325 | 0.238602 | 0.256508 | 0.267318 |
| *Acidobacteria* | 0.192842 | 0.071544 | 0.065210 | 0.026646 | 0.011258 | 0.015757 | 0.013060 | 0.012926 | 0.011191 |
| *Chloroflexi* | 0.093232 | 0.029936 | 0.029746 | 0.010967 | 0.005114 | 0.007308 | 0.005640 | 0.005551 | 0.005394 |
| *Verrucomicrobia* | 0.024049 | 0.013687 | 0.016652 | 0.007643 | 0.004488 | 0.006435 | 0.005898 | 0.009244 | 0.008595 |
| *Rokubacteria* | 0.040657 | 0.010922 | 0.008270 | 0.003223 | 0.001779 | 0.002574 | 0.002171 | 0.001712 | 0.001891 |
| *Patescibacteria* | 0.001623 | 0.001690 | 0.001007 | 0.000627 | 0.000313 | 0.007285 | 0.003727 | 0.007151 | 0.006782 |
| *Gemmatimonadetes* | 0.014503 | 0.004107 | 0.003816 | 0.001343 | 0.000560 | 0.001041 | 0.000593 | 0.000851 | 0.000683 |

**Table S2.** Relative abundance of the top 10 predominant bacteria in soil bacterial community at class level

| Class | CK | ATJ1 | AT1 | ATJ2 | AT2 | ATJ3 | AT3 | ATJ4 | AT4 |
| --- | --- | --- | --- | --- | --- | --- | --- | --- | --- |
| *Gammaproteobacteria* | 0.08316 | 0.036315 | 0.037736 | 0.27495 | 0.686799 | 0.232951 | 0.35933 | 0.301406 | 0.271884 |
| *Bacilli* | 0.006536 | 0.36638 | 0.407507 | 0.191667 | 0.128405 | 0.105116 | 0.132411 | 0.064191 | 0.080832 |
| *Bacteroidia* | 0.022997 | 0.003794 | 0.007778 | 0.114965 | 0.016898 | 0.296325 | 0.238591 | 0.256508 | 0.267172 |
| *Alphaproteobacteria* | 0.124007 | 0.083261 | 0.095918 | 0.094866 | 0.04351 | 0.130677 | 0.112357 | 0.178652 | 0.19545 |
| *Actinobacteria* | 0.093668 | 0.1713 | 0.122496 | 0.179704 | 0.057454 | 0.110746 | 0.063039 | 0.095627 | 0.084548 |
| *Thermoleophilia* | 0.089337 | 0.094832 | 0.097294 | 0.047103 | 0.020782 | 0.036259 | 0.026993 | 0.028839 | 0.027832 |
| *Subgroup_6* | 0.125618 | 0.055183 | 0.049374 | 0.018946 | 0.008147 | 0.010519 | 0.009087 | 0.008192 | 0.007397 |
| *Deltaproteobacteria* | 0.076098 | 0.0234 | 0.025403 | 0.011605 | 0.00611 | 0.018901 | 0.015063 | 0.016216 | 0.011829 |
| *Acidimicrobiia* | 0.054836 | 0.0343 | 0.032946 | 0.014671 | 0.00583 | 0.009792 | 0.00686 | 0.007744 | 0.008595 |
| *MB-A2-108* | 0.029936 | 0.034636 | 0.029432 | 0.009165 | 0.004174 | 0.006491 | 0.004521 | 0.003984 | 0.004588 |

**Table S3.** Relative abundance of the top 10 predominant bacteria in soil bacterial community at order level

| Order | CK | ATJ1 | AT1 | ATJ2 | AT2 | ATJ3 | AT3 | ATJ4 | AT4 |
| --- | --- | --- | --- | --- | --- | --- | --- | --- | --- |
| *Bacillales* | 0.006133 | 0.365787 | 0.407283 | 0.191287 | 0.128192 | 0.104904 | 0.132131 | 0.063967 | 0.07987 |
| *Pseudomonadales* | 0.002887 | 0.002104 | 0.003693 | 0.103897 | 0.634224 | 0.027362 | 0.163623 | 0.01945 | 0.064393 |
| *Rhizobiales* | 0.094362 | 0.067593 | 0.079959 | 0.078896 | 0.037646 | 0.090591 | 0.080205 | 0.117326 | 0.129983 |
| *Betaproteobacteriales* | 0.059771 | 0.020603 | 0.018577 | 0.093858 | 0.030473 | 0.098782 | 0.124331 | 0.134806 | 0.128797 |
| *Flavobacteriales* | 0.002663 | 0.000627 | 0.00282 | 0.027574 | 0.004767 | 0.167528 | 0.119004 | 0.122171 | 0.125126 |
| *Sphingobacteriales* | 0.00244 | 0.000414 | 0.003458 | 0.075718 | 0.009266 | 0.108955 | 0.096835 | 0.103494 | 0.101916 |
| *Xanthomonadales* | 0.002954 | 0.001365 | 0.002395 | 0.06446 | 0.005942 | 0.098279 | 0.05901 | 0.137313 | 0.065847 |
| *Corynebacteriales* | 0.028783 | 0.065221 | 0.080429 | 0.056984 | 0.032409 | 0.036281 | 0.032107 | 0.032051 | 0.035061 |
| *Gaiellales* | 0.051881 | 0.069238 | 0.069585 | 0.031469 | 0.01372 | 0.02274 | 0.016641 | 0.01776 | 0.016854 |
| *Micrococcales* | 0.011314 | 0.076613 | 0.010799 | 0.095884 | 0.010754 | 0.047953 | 0.010038 | 0.033808 | 0.008561 |

**Table S4.** Relative abundance of the top 10 predominant bacteria in soil bacterial community at family level

| Family | CK | ATJ1 | AT1 | ATJ2 | AT2 | ATJ3 | AT3 | ATJ4 | AT4 |
| --- | --- | --- | --- | --- | --- | --- | --- | --- | --- |
| *Pseudomonadaceae* | 0.002842 | 0.001701 | 0.003603 | 0.103147 | 0.627677 | 0.027284 | 0.161496 | 0.019013 | 0.063833 |
| *Bacillaceae* | 0.004902 | 0.22768 | 0.272824 | 0.068746 | 0.057566 | 0.053246 | 0.057051 | 0.042458 | 0.044473 |
| *Flavobacteriaceae* | 0.002305 | 0.000627 | 0.002753 | 0.02481 | 0.002428 | 0.164529 | 0.116016 | 0.12056 | 0.123145 |
| *Xanthomonadaceae* | 0.001891 | 0.00113 | 0.002205 | 0.064437 | 0.005931 | 0.098156 | 0.058954 | 0.137067 | 0.065724 |
| *Sphingobacteriaceae* | 0.000257 | 0.000313 | 0.003279 | 0.075572 | 0.009244 | 0.094787 | 0.086853 | 0.076434 | 0.071353 |
| *Mycobacteriaceae* | 0.027373 | 0.064214 | 0.079366 | 0.055093 | 0.032118 | 0.03524 | 0.03167 | 0.030999 | 0.034479 |
| *Burkholderiaceae* | 0.012411 | 0.007532 | 0.008326 | 0.038094 | 0.013519 | 0.050068 | 0.085779 | 0.064963 | 0.083171 |
| *Methyloligellaceae* | 0.016361 | 0.03167 | 0.039459 | 0.039247 | 0.021621 | 0.039594 | 0.039705 | 0.050997 | 0.05976 |
| *Subgroup_6* | 0.123671 | 0.054399 | 0.048569 | 0.018644 | 0.00808 | 0.010419 | 0.00902 | 0.008158 | 0.007319 |
| *Paenibacillaceae* | 0.000537 | 0.050337 | 0.050404 | 0.051501 | 0.034323 | 0.026724 | 0.044596 | 0.00883 | 0.01447 |

**Table S5.** Relative abundance of the top 10 predominant bacteria in soil bacterial community at genus level

| Genus | CK | ATJ1 | AT1 | ATJ2 | AT2 | ATJ3 | AT3 | ATJ4 | AT4 |
| --- | --- | --- | --- | --- | --- | --- | --- | --- | --- |
| *Pseudomonas* | 0.002842 | 0.001701 | 0.003603 | 0.103125 | 0.627677 | 0.027284 | 0.161496 | 0.019013 | 0.063811 |
| *Bacillus* | 0.004779 | 0.206764 | 0.254157 | 0.060823 | 0.052732 | 0.049844 | 0.05375 | 0.040701 | 0.042626 |
| *Flavobacterium* | 0.002305 | 0.000627 | 0.002753 | 0.02481 | 0.002428 | 0.164529 | 0.116005 | 0.12056 | 0.123145 |
| *Pedobacter* | 2.24E-05 | 1.12E-05 | 0.002999 | 0.067146 | 0.00789 | 0.092202 | 0.080452 | 0.07452 | 0.068231 |
| *Mycobacterium* | 0.027373 | 0.064214 | 0.079366 | 0.055093 | 0.032118 | 0.03524 | 0.031648 | 0.030999 | 0.034479 |
| *Lysobacter* | 0.000895 | 0.000671 | 0.001287 | 0.058327 | 0.003111 | 0.091855 | 0.036841 | 0.12394 | 0.053728 |
| *Subgroup_6* | 0.123671 | 0.054399 | 0.048569 | 0.018644 | 0.00808 | 0.010419 | 0.00902 | 0.008158 | 0.007319 |
| *Methylotenera* | 0.000291 | 3.36E-05 | 0.000504 | 0.048927 | 0.013832 | 0.043555 | 0.033987 | 0.062401 | 0.038463 |
| *Paenisporosarcina* | 0.000313 | 0.073301 | 0.06634 | 0.048792 | 0.016518 | 0.010922 | 0.006905 | 0.004409 | 0.002932 |
| *uncultured* | 0.010273 | 0.022483 | 0.027496 | 0.026399 | 0.014235 | 0.024844 | 0.024631 | 0.030954 | 0.036807 |

**Table S6.** Information of sequencing parameters for different samples

| Sample | Input | Filtered | Denoised | Merged | Non-chimeric | Non-singleton |
| --- | --- | --- | --- | --- | --- | --- |
| CK_1 | 72140 | 68513 | 60052 | 43538 | 37367 | 31354 |
| CK_2 | 77128 | 68038 | 63809 | 51866 | 45711 | 43910 |
| CK_3 | 72382 | 63478 | 58950 | 46108 | 40071 | 38313 |
| ATJ1_1 | 71351 | 67985 | 64574 | 57959 | 43753 | 42249 |
| ATJ1_2 | 71034 | 67835 | 63789 | 55682 | 41651 | 39464 |
| ATJ1_3 | 71004 | 66694 | 63563 | 57314 | 47582 | 46298 |
| AT1_1 | 68595 | 65134 | 61682 | 54551 | 39344 | 37526 |
| AT1_2 | 70226 | 66428 | 63095 | 56132 | 45112 | 43606 |
| AT1_3 | 66902 | 63423 | 60421 | 54279 | 43690 | 42417 |
| ATJ2_1 | 95104 | 85611 | 81901 | 73113 | 60881 | 59303 |
| ATJ2_2 | 76875 | 73326 | 70492 | 64568 | 52803 | 51448 |
| ATJ2_3 | 70686 | 67399 | 64416 | 58634 | 46962 | 45591 |
| AT2_1 | 69676 | 66561 | 64822 | 61943 | 53598 | 53013 |
| AT2_2 | 63152 | 59411 | 57683 | 54459 | 45769 | 45052 |
| AT2_3 | 73203 | 69769 | 67872 | 64967 | 56123 | 55333 |
| ATJ3_1 | 72801 | 69851 | 67105 | 61159 | 48652 | 47411 |
| ATJ3_2 | 73857 | 70566 | 67857 | 61907 | 51043 | 49817 |
| ATJ3_3 | 71758 | 67881 | 65571 | 60722 | 53241 | 52501 |
| AT3_1 | 69747 | 66395 | 64064 | 59677 | 49426 | 48587 |
| AT3_2 | 66813 | 64037 | 61472 | 56965 | 45494 | 44320 |
| AT3_3 | 74997 | 71576 | 69049 | 63726 | 51835 | 50510 |
| ATJ4_1 | 70521 | 66963 | 64179 | 58140 | 48225 | 47028 |
| ATJ4_2 | 71049 | 67753 | 64955 | 58682 | 45689 | 44282 |
| ATJ4_3 | 67622 | 64763 | 62046 | 56461 | 46637 | 45488 |
| AT4_1 | 67825 | 64783 | 62079 | 56482 | 45180 | 43972 |
| AT4_2 | 81379 | 76683 | 73485 | 66366 | 53897 | 52662 |
| AT4_3 | 96775 | 87290 | 81390 | 65988 | 49411 | 46324 |

Note: The second column is the amount of original data, the third column is the amount of data after removing the low-quality sequence, the fourth column is the amount of sequence data after denoising, the fifth column is the sequence quantity after splicing, the sixth column is the sequence quantity after removing the chimera, and the seventh column is the sequence quantity after removing singleton.
